# The Effect of Intravenous Lidocaine on Trigeminal Neuralgia: A Randomized Double Blind Placebo Controlled Trial

**DOI:** 10.1155/2014/853826

**Published:** 2014-03-10

**Authors:** Evmorfia Stavropoulou, Erifili Argyra, Panagiotis Zis, Athina Vadalouca, Ioanna Siafaka

**Affiliations:** ^1^1st Anaesthesiology Clinic, Pain Relief and Palliative Care Unit, Aretaieion University Hospital, 76 Vas. Sofias Avenue, 11527 Athens, Greece; ^2^Department of Neurology, Evangelismos General Hospital, 45-47 Ipsilantou Street, 10676 Athens, Greece

## Abstract

Trigeminal neuralgia is the most common neuralgia. Its therapeutic approach is challenging as the first line treatment often does not help, or even causes intolerable side effects. The aim of our randomized double blind, placebo controlled, crossover study was to investigate in a prospective way the effect of lidocaine in patients with trigeminal neuralgia. Twenty patients met our inclusion criteria and completed the study. Each patient underwent four weekly sessions, two of which were with lidocaine (5 mgs/kg) and two with placebo infusions administered over 60 minutes. Intravenous lidocaine was superior regarding the reduction of the intensity of pain, the allodynia, and the hyperalgesia compared to placebo. Moreover, contrary to placebo, lidocaine managed to maintain its therapeutic results for the first 24 hours after intravenous infusion. Although, intravenous lidocaine is not a first line treatment, when first line medications fail to help, pain specialists may try it as an add-on treatment. This trial is registered with NCT01955967.

## 1. Introduction

Trigeminal neuralgia (TN) is the most common neuralgia, with an annual incidence of 5/100000 [1]. The International Association for the Study of Pain (IASP) defines TN as sudden, usually unilateral, severe, brief, stabbing, and recurrent episodes of pain in the distribution of one or more branches of the trigeminal nerve.

Treatment guidelines, published from the American Academy of Neurology (AAN) and the European Federation of Neurologic Societies (EFNS), recommend carbamazepine or oxcarbazepine as the first choice pharmacological treatment of TN and baclofen or lamotrigine as the second choice [2]. However, some patients may experience intractable pain despite adequate treatment with these medications or their combination. On the other hand, some patients may experience intolerable side effects that lead to discontinuation, although recommended treatments have achieved sufficient reduction of their pain.

Lidocaine is a common amino amide-type local anesthetic and antiarrhythmic drug [3]. It is mainly used to relieve cancer or postoperative pain [4, 5], however, it has also been used to relieve several kinds of neuropathic pain, including postherpetic neuralgia [6] and intractable TN [7, 8]. This therapeutic potential lies in the fact that systemic lidocaine and its oral congeners can block sodium channels in a dose dependent fashion [9, 10] in both the peripheral and the central nervous system [11].

In the literature, only few retrospective studies of the effect of intravenous lidocaine on TN exist. The aim of our randomized double blind, placebo controlled, crossover study was to investigate in a prospective way the effect of lidocaine in patients with TN.

## 2. Materials and Methods

### 2.1. Participants

Following the approval of the Institutional Ethical Committee of Aretaieion Hospital, University of Athens, all consecutive patients suffering from TN who visited the Outpatient Pain and Palliative Care Center were invited to participate in the study.

To be enrolled, the patients had to meet the following inclusion criteria: (1) confirmed diagnosis of TN according to IASP definition, (2) age equal to or greater than 18 years, (3) visual analogue scale (VAS) score equal to or greater than 3 (out of a maximum 10), (4) Douleur Neuropathique 4 Questionnaire (DN4) score equal to or greater than 4 (out of a maximum 10), (5) having received the recommended medications for TN (antiepileptics, spasmolytics, opioids, anti-inflammatory, and simple analgesic drugs) for an adequate period without therapeutic results, (6) TN duration of at least 12 months, (8) no history of allergy to lidocaine, (9) no history of substance abuse, (10) absence of severe psychiatric diseases, (11) not being pregnant, (12) not lactating, (13) absence of severe cardiac, hepatic, and renal decease, and (14) be willing to provide a written informed consent to undergo the experimental procedures.

### 2.2. Procedures

Each patient participated in four sessions, every 2nd day, receiving two active and two placebo treatments, involving continuous infusion of 1 hour. Active treatment was lidocaine (5 mg per kilogram of body weight) in 250 mL of 5% dextrose solution and placebo treatment was 250 mL of 5% dextrose solution.

Subjects were randomly assigned to treatment sequence via a computer-generated randomization list. A randomization list was prepared in sealed envelopes for each patient. Investigator A was responsible for solution preparation according to the randomization list. Investigator B, who was blind to the treatment, was responsible for clinical examination and treatment administration. At the end of each treatment investigator B had to record data and enclose the forms in envelopes that remained closed until the end of the study.

In the morning of each session (08:00) the patients were guided to a calm and separate room where investigator B recorded the pain intensity on VAS before the beginning of the infusion and at the end of it. Moreover, investigator B tested the intensity of (1) mechanical allodynia (pressure of the painful area by an unused pencil), (2) thermal allodynia (application of a thermal roll at 40°C at the painful area), (3) cold allodynia (application of a thermal roll at 26°C at the painful area), (4) pinprick hyperalgesia (feeling excess pain more than usual pain during the pinprick by a needle 26G at the painful area), (5) hot hyperalgesia (application of a thermal roll at 46°C at the of the painful area), and (6) cold hyperalgesia (application of a thermal roll at 20°C at the painful area).

During the sessions the patients were monitored continuously by 3-lead electrocardiogram, pulse oximetry, and blood pressure (BP) manometer. Every 15 minutes systolic BP, diastolic BP, oxygen saturation, and heart rate (HR) were recorded. Any side effects, such as somnolence, mental confusion, metal taste, tingling sensation around the mouth, vision disturbances, tremor, drowsy mouth, tremor, or anything else were also documented.

Finally, at the end of each session the patients were given a “Pain Diary,” where they recorded the VAS score at 16:00, 20:00, and 24:00 the same day and at 08:00 the next morning.

### 2.3. Statistical Analyses

A database was developed using the Statistical Package for Social Science (version 16.0 for Mac; SPSS). Frequencies and descriptive statistics were examined for each variable. Comparisons between patients who received lidocaine and patients who received placebo were made using Student's *t*-tests for normally distributed continuous data and Mann-Whitney's *U* test for nonnormally distributed data.

Allodynia and hyperalgesia were graded according to a Likert scale from 0 to 4 (0 = no pain, 1 = little pain, 2 = moderate pain, 3 = heavy pain, and 4 = unbearable pain). Grading scores in allodynia and hyperalgesia before and after intravenous administration were recorded. Patients who achieved a reduction of at least 1 grading point in the Likert scale were considered to have achieved a reduction of allodynia and hyperalgesia, when patients who either graded the same before and after or showed increase in the Likert scale were considered not to have achieved a reduction. Comparisons between the lidocaine and placebo groups regarding these categorical data were made using the chi-square test.

Power analysis demonstrated that a 20% difference in VAS reduction with 80% power could be detected with a sample of 20 patients.

A value of *P* < 0.05 was considered to be statistically significant.

## 3. Results

### 3.1. Study Population

Between February 2007 and September 2011, 23 individuals fulfilled the above-mentioned inclusion criteria. However, three patients denied continuing after the first session. Therefore, our final study population consisted of twenty patients. Clinical and demographic characteristics of the study total population are summarized in Table 1.

### 3.2. Response to Treatment

**Figure 1:**
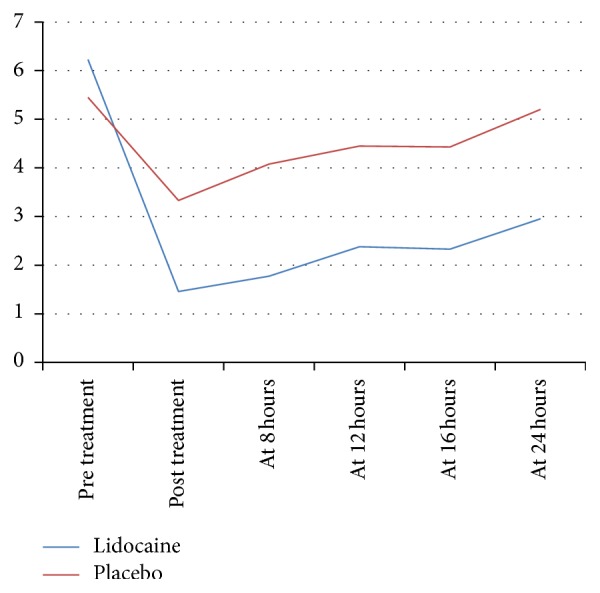
Intensity of pain according to VAS score (vertical axis), during the first 24 hours after treatment (horizontal axis) between lidocaine (5 mg per kilogram of patient's weight) and placebo treatment groups.

Table 2 shows the response regarding intensity of pain, allodynia, and hyperalgesia following lidocaine versus placebo intravenous infusion. Both lidocaine and placebo reduced the intensity of pain, at the end of each session; however, lidocaine achieved a greater reduction compared to placebo (76.4% versus 40.1%, *P* < 0.001). This was maintained for at least 24 hours after treatment as was documented in the pain diary. Hence, at 24 hours after treatment lidocaine achieved a reduction of 52% of the pretreatment intensity of pain when patients who received placebo reported a mild increase of 4% of the pretreatment intensity of pain (Figure 1).

Similarly, lidocaine reduced mechanical allodynia thermal allodynia, cold allodynia, pinprick hyperalgesia, hot hyperalgesia, and cold hyperalgesia in a statistically significant greater percentage of patients.

### 3.3. Adverse Events

No statistically significant changes regarding systolic BP, diastolic BP, HR, and oxygen saturation were found during the treatment administration between patients who received lidocaine and patients who received placebo.

Table 3 summarizes the adverse events as reported by the patients after each treatment. All side effects were reported as mild. Patients who received lidocaine reported more side effects compared to patients who received placebo. Most common side effect among patients who received lidocaine was somnolence (reported in 32.5% of cases) and most common side effect among patients who received placebo was dry mouth (reported in 5% of cases).

## 4. Discussion

Intravenous lidocaine infusions are gaining acceptance in a variety of pain-management settings [12]. This double blind, randomized, crossover, placebo controlled study aimed to investigate the effect of intravenous lidocaine on trigeminal neuralgia. We showed that intravenous lidocaine is superior regarding the reduction of the intensity of pain, the allodynia, and the hyperalgesia compared to placebo and that lidocaine managed to maintain its therapeutic results during the first 24 hours after intravenous infusion.

To our knowledge, this is the first prospective study of the effect of intravenous lidocaine on trigeminal neuralgia compared to placebo. Moreover, in a nonclinical setting not many studies examining the alteration of the evoked pain of TN by the intravenous administrations of lidocaine exist. An experimental study in neuropathic rats had already shown that intravenous, but not intrathecal or regionally applied, lidocaine produces dose dependent suppression allodynia associated with nerve injury. Interestingly enough, the effects far outlast plasma concentrations of lidocaine; however, the mechanism of these prolonged effects remains unknown [13]. In our study we also observed that lidocaine achieved a decrease of the intensity of pain, as this was measured by VAS, which lasted for 24 hours and this decrease was superior compared to placebo, despite the fact that lidocaine's half-life is 1,6 hours [14]. Similar to this, Tremont-Lukats et al. reported that the effect of lidocaine in patients with neuropathic pain started 4 hours after the onset of treatment and continued for at least 4 hours after the end of the infusion [15]. Moreover, Attal et al. showed that, in patients with central pain, lidocaine decreased VAS for 6 hours after the injection and a subgroup of patients experienced prolonged analgesia for up to 7 days [16]. Furthermore, Arai et al. claimed that in some patients who suffered from trigeminal neuralgia and had pain relief after receiving lidocaine and magnesium the therapeutic result lasted for almost one year [7].

Until now, the effectiveness of lidocaine in neuropathic pain has been shown through case series. Khawaja et al. in a case series concluded that the use of 5% lidocaine plasters may be a useful adjunctive tool for the management of chronic orofacial pain. Interestingly, several patients in this study commented that the plasters significantly assisted breakthrough pain, particularly cold allodynia caused by exposure of the face to cold air resulting in excruciating pain [17]. Moreover, Arai et al. have recently published data of nine patients with TN treated with an intravenous infusion of a combination of 1.2 g magnesium and 100 mg lidocaine for 1 hour, once a week for 3 weeks. The authors concluded that all patients experienced sound pain relief after the combined intravenous infusion therapy [7]. However, as their study was performed in a retrospective way, the authors did not use a control group. The authors explain that because of the very low incidence of intractable TN it is impractical to perform a randomized placebo controlled trial.

As we also had difficulty in identifying a large sample of patients to be recruited in the form of a randomized placebo controlled trial we performed a crossover trial. Thus, each patient participated in four sessions, receiving two active and two placebo treatments. The assignment to treatment sequence was random. This is a very good alternative way to study different drug types and/or doses when the study sample is small [18]. One other advantage of our study is that we used a standardized dose of lidocaine according to the patients' weight. We chose to use the dose of 5 mg/kgr lidocaine over 60 minutes because in this dose lidocaine does not affect the peripheral conduction [19, 20] and it, also, acts at hyperexcitable neurons without affecting normal nerve conduction showing, thus, good effects on neuropathic pain [21].

Regarding its safety profile, intravenous lidocaine has been used to relieve several kinds of neuropathic pain without producing major adverse effects [22]. Similarly, in our study, the infusions caused minor side effects and during the infusions all the patients were haemodynamically stable with good oxygen saturation. Moreover, no dropouts were observed because of the occurrence of side effects.

Finally, our results should be interpreted with some caution given the fact that we only used a single dose of lidocaine (5 mg/kg). Therefore the effectiveness of different doses remains unstudied. Future clinical research should focus on identifying the least effective dosage of intravenous lidocaine.

Intractable TN remains a difficult to manage type of neuropathic pain. Interventional procedures, such as microsurgical decompression, have good results in many patients [2]. However, such procedures involve the risk of major neurological complications and other less serious adverse effects and their result may not last. Pharmaceutical approach still remains the mainstay of treatment. Although intravenous lidocaine is not a first line treatment, when first line medications fail to help, pain specialists may try it as an add-on treatment.

## Figures and Tables

**Table 1:** Characteristics of the study population (*n* = 20).

Demographic characteristics	
Male sex (%)	7 (35)
Age in years (SD)	65.20 (15.28)
Clinical characteristics	
Weight in kg (SD)	73.75 (16.48)
Location of neuralgia (%)	
1st trigeminal branch	2 (10)
2nd trigeminal branch	2 (10)
3rd trigeminal branch	16 (80)
DN4 score at baseline (SD)	6.25 (0.63)

DN4: Douleur Neuropathique 4 Questionnaire; SD: standard deviation.

**Table 2:** Response regarding intensity of pain, allodynia, and hyperalgesia following lidocaine versus placebo intravenous infusion. The study included 20 patients who received twice the active (lidocaine) and twice the placebo drug.

	Lidocaine	Placebo	*P*
	(*n* = 40)	(*n* = 40)	
Effect on intensity of pain			
VAS score pretreatment (SD)	6.23 (1.56)	5.45 (2.04)	0.060
VAS score posttreatment (SD)	1.46 (1.37)	3.33 (2.02)	**<0.001 **
VAS score at 16:00 (SD)	1.77 (1.61)	4.08 (2.33)	**<0.001 **
VAS score at 20:00 (SD)	2.38 (1.73)	4.45 (2.36)	**<0.001**
VAS score at 24:00 (SD)	2.33 (1.84)	4.43 (2.15)	**<0.001 **
VAS score at 08:00 next morning (SD)	2.95 (1.88)	5.20 (2.55)	**<0.001**
VAS reduction % pre-/posttreatment (SD)	76.4 (23.0)	40.1 (31.9)	**<0.001**
VAS reduction % pretreatment—16:00 (SD)	70.5 (27.7)	21.6 (45.5)	**<0.001**
VAS reduction % pretreatment—20:00 (SD)	59.5 (30.3)	20.2 (42.1)	**<0.001**
VAS reduction % pretreatment—24:00 (SD)	61.0 (32.1)	13.9 (39.1)	**<0.001**
VAS reduction % pretreatment—next day (SD)	52.0 (29.6)	−4.0 (56.3)	**<0.001**
Effect on allodynia			
Reduced mechanical allodynia (%)	31 (77.5)	20 (50.0)	**0.011**
Reduced thermal allodynia (%)	18 (45.0)	7 (17.5)	**0.008**
Reduced cold allodynia (%)	15 (37.5)	5 (12.5)	**0.010**
Effect on hyperalgesia			
Reduced pinprick hyperalgesia (%)	26 (65.0)	14 (35.0)	**0.007**
Reduced hot hyperalgesia (%)	22 (55.0)	7 (17.5)	**<0.001**
Reduced cold hyperalgesia (%)	20 (50.0)	11 (27.5)	**0.039**

VAS: visual analogue scale; SD: standard deviation.

**Table 3:** Adverse events reported by patients. The study included 20 patients who received twice the active lidocaine and twice the placebo drug.

	Lidocaine	Placebo
	(*n* = 40)	(*n* = 40)
Somnolence	13	1
Dry mouth	5	2
Dizziness	5	0
Headache	3	1
Feeling flushed	2	0
Confusion	1	0
Dysarthria	1	0
Tinnitus	1	0
